# Artificial intelligence-informed planning for the rapid response of hazard-impacted road networks

**DOI:** 10.1038/s41598-022-19637-z

**Published:** 2022-09-29

**Authors:** Li Sun, John Shawe-Taylor, Dina D’Ayala

**Affiliations:** 1 Department of Civil, Environmental & Geomatic Engineering, University College London, London, WC1E 6BT UK; 2 Department of Computer Science, University College London, London, WC1E 6BT UK; 3 International Research Centre on Artificial Intelligence (IRCAI), Institute Jozef Stefan, Ljubljana, 1000 Slovenia

**Keywords:** Civil engineering, Computational science

## Abstract

Post-hazard rapid response has emerged as a promising pathway towards resilient critical infrastructure systems (*CISs*). Nevertheless, it is challenging to scheme the optimal plan for those rapid responses, given the enormous search space and the hardship of assessment on the spatiotemporal status of *CISs*. We now present a new approach to post-shock rapid responses of road networks (*RNs*), based upon lookahead searches supported by machine learning. Following this approach, we examined the resilience-oriented rapid response of a real-world *RN* across *Luchon*, *France*, under destructive earthquake scenarios. Our results show that the introduction of one-step lookahead searches can effectively offset the lack of adaptivity due to the deficient heuristic of rapid responses. Furthermore, the performance of rapid responses following such a strategy is far surpassed, when a series of deep neural networks trained based solely on machine learning, without human interventions, are employed to replace the heuristic and guide the searches.

## Introduction

The modus operandi of modern urban systems is increasingly contingent upon the enduring functionality of an array of critical infrastructure systems (*CISs*), which are becoming increasingly sophisticated and interconnected, in response to societal demands^1,2^. Therefore, resilience of those *CISs* will be strategically crucial to the well-being of urban systems under disruptive events, such as natural hazards and pandemics, etc. Conceptually, *CIS* resilience has been widely characterized as their capacity to absorb the effects of a disruption on their performance, dependent on their robustness, and restore that performance, in a prompt and sound pattern^3^. Nonetheless, many of those *CISs* that rely on components and sub-systems developed sometimes over centuries have proven to be inadequately resilient to those disruptive events, and significant death toll, as well as socio-economic losses can be thus triggered^4^. In particular, statistical studies conducted by the *United Nations* have revealed that, earthquake-induced death toll accounts for nearly 60% of the global fatalities from disasters, throughout the past decades^5^.

One of the root causes identified for the lack of *CIS* resilience lies in the inability of state-of-the-art disaster management systems to deliver expeditious and effective recoveries in the immediate aftermath of hazards, which could significantly exacerbate earthquake-initiated losses. Against this backdrop, recent studies have revealed that, instead of the indiscriminate characterization on the criticality associated with different time-windows throughout catastrophes, the minimization of functionality losses of *CISs*, through rapid responses in the immediate aftermath should be explored as a new, promising blueprint for future resilient *CISs*^6^. In this respect, hazard resilience of the road network (*RN*) in an exposed region is a critical requisite, as it underpins the mobility in the wake of damaging events such as earthquakes, and can therefore hinder the recovery of other *CIS*, whose damaged components cannot be accessed, and hence prompt repairs cannot be delivered^7–9^. In view of such a criticality, *RNs* are the focus of this study. Nevertheless, it will be highlighted that, given the dimensions of the state space of real-world *CISs* like *RNs*, it remains challenging to scheme an “optimal” plan of the post-hazard rapid response campaign in an expeditious pattern using conventional stochastic optimization routines^10,11^.

To address such a challenge, we present different approaches to the effective and viable strategy of post-shock rapid response of real-world *RNs* subject to earthquake hazards, in this study. Initially, *heuristic-based strategies* are considered, based on simple ranking criteria. However, such strategies show limited adaptability when realistic topology of *RNs* and actual damage status are considered. Hence, based upon those heuristics, a *lookahead-based strategy* is introduced to deliver the decision-making on the rapid response in a recursive and more adaptive way^12^. Notwithstanding its benefits, the lookahead search approach proves to be computationally costly, and thus potentially infeasible, when large *RNs* are considered. To overcome such an obstacle, a *learning-based strategy*, which marries the lookahead search with *deep reinforcement learning*^13,14^, is proposed. In such a strategy, by learning the “experiences” generated from a large amount of hypothetical, different earthquake/damage scenarios, a deep neural network is trained and employed to guide the search, bypassing the need of iterative simulations associated with the *lookahead-based strategy*. In particular, to examine the generalization capacity of the *learning*, an initial (1st generation) neural network is purposely trained based upon the experiences associated with a naïve heuristic of rapid responses, whereupon no sophisticated domain knowledge is imparted. The dataset of experiences generated by this neural network are then employed to train the subsequent generations, each of which is expected to yield a better training dataset for the next one. In practice, there is a limit to the performance improvement of such a *learning-based strategy*, which can be reached after a few recurrences (see “Methods” section).

To measure the planning capability of the *learning-based strategy*, we apply the three different strategies described above to a real-world, regional-scale *RN* in *Luchon*, *France*, affected by catastrophic earthquake scenarios. Our study consistently shows that the introduction of lookahead searches can significantly compensate for the lack of adaptivity of post-shock rapid responses driven by heuristic-based strategies. With regard to the third strategy, without domain knowledge instilled, the *deep reinforcement learning-oriented* neural network trained following the pipeline proposed in this research is found to enable an increasingly accurate assessment of the global damage status of the *RN*, in an autonomous way. Correspondingly, performance of the rapid response guided by such a strategy is demonstrated to outperform the two previous ones, by better accounting for the interdependence between the concurrent rapid responses and full repairs. Hence, findings in this paper can be leveraged to inform the decision-making of the resilience-oriented rapid response of real-world infrastructure systems under damaging events. Besides, owing to its scalability and adaptivity, the modelling framework can be employed to shed light on risk governance of large-scale, globally networked socio-economic systems, where interconnected dependences are ubiquitous^15–17^.

## Results

### Earthquake hazard scenarios

**Figure 1:**
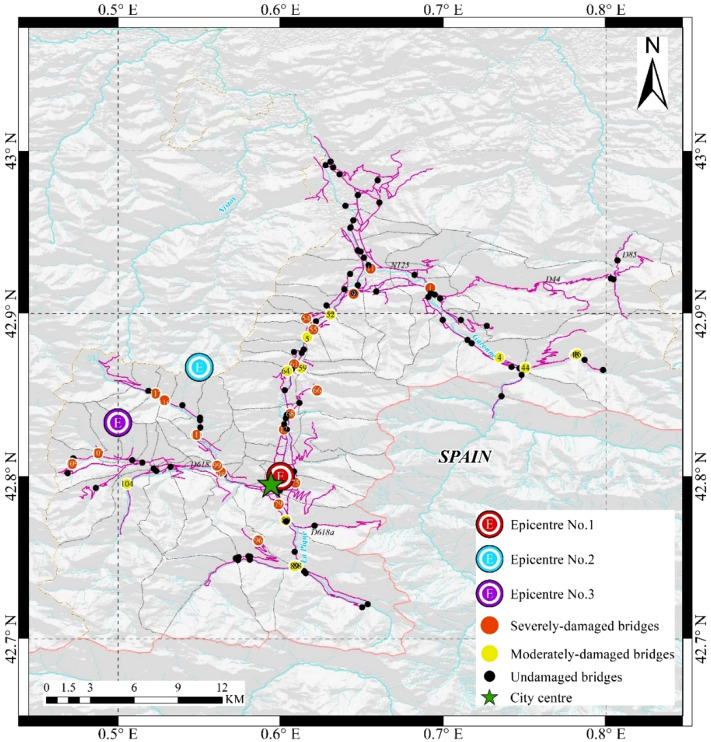
Topology of the *Road Network* in *Luchon*, *France*. The map shows the location of the three Epicentres included in this study. The damage state of the global *RN* is obtained by conducting seismic fragility analysis^19–21^, under the earthquake scenario with *Epicentre No.* 1 and magnitude of 7, as the maximum magnitude obtained by the source area model across the region^22^. The figure is plotted by ArcGIS.

*Luchon* is a well-known historic and touristic region, located in one of the valleys of *Pyrenees* connecting *France* to *Spain*. Despite its relatively small size, the *road network* embedded in *Luchon* is hierarchical, given the different tier of the routes included, namely, the national, departmental and rural ones. Such a network is also sufficiently heterogenous, given the radically-different span and seismic fragility of the 118 bridges present along its segments. Based on the recorded seismic activities across the region, three epicentres close to the central area of *Luchon*, which are thus expected to induce the most widespread damage, have been chosen in this study^18^ (see Fig. 1).

### Damage assessment

Our methodology assesses the impact of those scenarios on the system-level, physical functionality status of the *RN*, by determining the damage state of each individual bridge through fragility analysis (see Supplementary Table S2). Without losses of generality, representative damage scenarios are obtained through the realization of 1000 Monte Carlo (*MC*) simulations of the *RN* under seismic scenario of *Epicentre No.* 1 and magnitude of 7, one of which is presented in Fig. 1, showing the generated geographic distribution of bridges with moderate and severe damage, respectively.

### Post-shock rapid responses guided by heuristic-based strategies

We firstly examine the behaviour of rapid responses following the *span-* and *betweenness-based* heuristics, respectively, under the seismic scenario discussed above. In principle, the *span-based* heuristic^6^, which prioritizes those bridges with shorter spans (which is a straightforward indicator of the repairability of bridges), is attempting to maximize the number of severely-damaged bridges being partially repaired in a short time frame after the main shock. By comparison, the *betweenness-based* heuristic is governed by the restoration of the functionality of those bridges, which are most critical to the global connectivity of the *RN*. Based upon the multi agent-based model (*ABM*) developed (see “Methods” section), rapid response will be delivered to severely-damaged bridges by *Agent A*, while the full repair will be delivered to moderately-damaged bridges, by *Agent B*.

**Figure 2:**
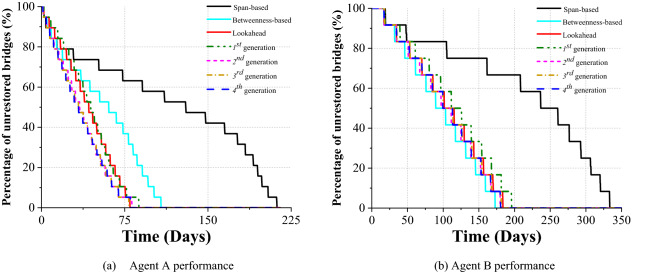
Time-varying, median number of unrestored bridges throughout the rapid response campaign, under different guiding strategies proposed. This plot tracks the median behaviour of the repair agent of severely- and moderately-damaged bridges, respectively, pursuant to 1000 simulations run. By doing so, their overall performance can be benchmarked against each other, and therefore highlighted. (**a**) The proportion of severely-damaged bridges, remaining to receive rapid response delivered by *Agent A* following each strategy. (**b**) The proportion of moderately-damaged bridges, remaining to be fully repaired by *Agent B*, with respect to each of the strategy adopted by *Agent A*.

Testing on the set of *MC* simulations, the median proportion of severely damaged bridges awaiting rapid response is tracked in Fig. 2a. It can be found that, the trajectories associated with those two distinct heuristics start to diverge on the 18th day after the shock, notwithstanding the same behavioural attributes of the agents (see Table 2). For the *span-based* heuristic, the series of long plateaux reveal that the rapid response is encumbered by the presence of the other damaged bridges (see “Methods” section), so the complete delivery of rapid responses takes 212.25 days. By comparison, the *betweenness-based* heuristic, by targeting different repair sequences, shortens such a duration to 107.75 days, a reduction of 49.2%.

In Fig. 2b, the restoration of moderately damaged bridges, delivered by *Agent B*, is tracked. Such an endeavour is also found to be substantially affected by the two different heuristics adopted by *Agent A*, due to their interdependence. In the case of the *span-based* heuristic, the full repair campaign turns out to be significantly stalled by the not-yet repaired severely-damaged bridges. Until the end of the 7th month after the shock, which is approximately the end of the concurrent rapid response campaign, the full-repair of each moderately-damaged bridge takes on average 55 days, substantially longer than the expected time, given the scope of the efficiency attribute of such an agent (namely, *E*_*m*_), as shown in Table 2. The repair of the remaining bridges speeds up thereafter, and is eventually completed on the 333.5th day. Such an outcome highlights that the *span-based* heuristic will not only lead to stagnant rapid response endeavours, it will also fail to bolster the contemporaneous full repairs, given its significant lack of adaptivity, as well as the absence of the coordination between the two campaigns. However, when the *betweenness-based* heuristic is adopted by *Agent A*, the full repair has been substantially improved, and is finalized in 172.75 days, 51.8% of that needed by the *span-based* heuristic. Such results have highlighted the profound impact of the topological configuration of large-scale infrastructure systems on the post-hazard recovery, and the critical role of appropriate rapid response strategies.

Given the significant uncertainty associated with the seismic damage and successive recovery of *RNs*, it may be insufficient to use the median performance as the sole indicator of the performance of different strategies. It is important to consider that when an earthquake strikes, we would like to be assured the strategy is likely to work well for that particular damage scenario. The median tells us that there is a 50% chance it will take less than that time. In order to have higher confidence in our estimates, higher conditional quantiles of the behaviour of the post-shock rapid response are also employed to measure the performance of the array of strategies developed in this research. Specifically, 75%- and 95%-quantile of the duration of the rapid response campaign for all those strategies are compiled in Table 1. The drop of the 75%-quantile of the duration of the *betweenness-based* heuristic can be found to reach 61.3%, compared to the corresponding outcome of the *span-based* one.

**Table 1:** Performance of rapid responses guided by different strategies.

	Span-based		Betweenness-based (baseline)		Lookahead-based		1st generation		2nd generation	
	Duration (days)	GWL (10^3^)	Duration (days)	GWL (10^3^)	Duration (days)	GWL (10^3^)	Duration (days)	GWL (10^3^)	Duration (days)	GWL (10^3^)
Median	212.25	2.86	107.75	1.21	79.75	1.01	87.75	1.13	81.5	1.00
75%-quantile	343	4.96	132.75	1.74	99	1.43	109.75	1.68	99.5	1.37
95%-quantile	360	8.15	184	2.95	136.63	2.47	169	3.16	134.75	2.27

To better understand the overall impact of different rapid response strategies on the network performance, besides the duration of the whole campaign, we further introduce the gross weighted losses (*GWLs*), which measure the system-level recovery state of *RNs* by aggregating their connectivity losses, throughout the whole rapid response campaign (see “Methods” section).

**Figure 3:**
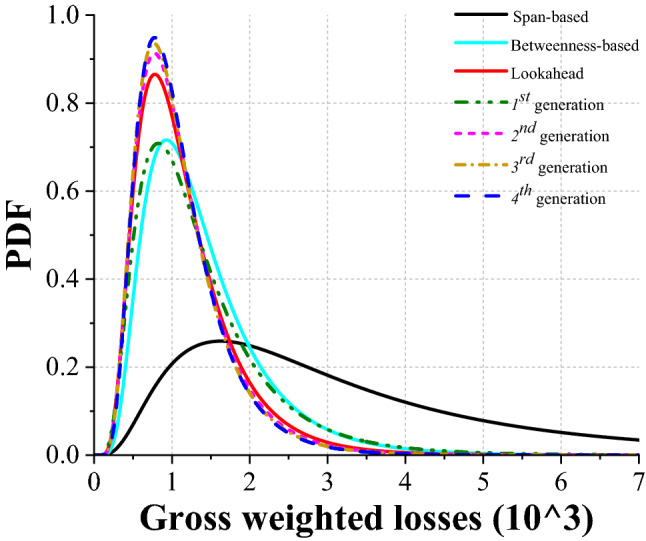
Probabilistic distribution of *GWLs* under scenario with epicentre *No.* 1 and magnitude of 7. 1000 simulations have been run for the rapid response under each of the different strategies. Accordingly, probabilistic density functions (*PDF*) has been fitted based to the dataset obtained from those simulations. The contrast among the behaviour of the rapid response according to those strategies as measured by *GWL*, is thus illustrated.

Based on the fitted probabilistic density functions (*PDFs*) following lognormal distribution presented in Fig. 3 (the comparison between the simulation outcomes and the corresponding fittings is presented in Supplementary Table 1), the median, 75%- and 95%-quantile of the *GWL* with regard to the *betweenness-based* heuristic, from which the rapid response and the full repair both benefits, yields a 57.7%, 64.9% and 63.8% reduction, respectively, compared to the corresponding outcome of the *span-based* heuristic.

### Post-shock rapid responses guided by the lookahead-based strategy

We examined the behaviour of the rapid response based upon the lookahead-based strategy (see “Methods” section), under the same earthquake scenario. It is noteworthy that, the one-step lookahead is followed by the *span-based* heuristic by design, which has already been demonstrated to be substantially less effective than the *betweenness-based* one. By doing so, the competitive edge of such a new strategy can be examined, with regard to the *betweenness-based* heuristic, herein considered as the performance *baseline*.

As shown in Fig. 2a, driven by the lookahead search, the rapid response is completed by the 79.75th day following the shock, corresponding to just 37.6% of the duration regarding the pure *span-based* heuristic. When compared to the *baseline*, despite trailing in the initial stage, the *lookahead-based* strategy starts outperforming, from the 31st day after the shock. Specifically, it can be observed that the trajectory associated with such a strategy has an almost-constant slope throughout the entire rapid response campaign, obtaining a 26.0% reduction in the time to finalize.

On the other hand, it should be noted that, the concurrent full repair is found to benefit less from the *lookahead-based* strategy, compared to the *betweenness-based* one (Fig. 2b), as it takes 183.75 days to complete the repair of all the moderately-damaged bridges, longer than the 172.75 days of the baseline strategy. Nonetheless, in terms of the *GWL*, such a strategy results in a median and 75%-quantile decrease of 16.5% and 17.8%, respectively, compared to the baseline. Moreover, the corresponding 95%-quantile is also found to be 16.3% lower, indicating that the lookahead strategy outperforms the baseline substantially, even in extreme cases (Table 1).

### Post-shock rapid responses guided by the learning-based strategy

We firstly trained the 1st generation Deep Neural Network (*DNN*), based upon the dataset of “experiences” generated following the *span-based* heuristic, which has been applied to 8000 random earthquake scenarios (see “Methods” section). Accordingly, we further investigated whether the rapid response driven by the lookahead search contingent upon the *DNN* can match or approximate the behaviour associated with the *lookahead-based* strategy with the *span-based* heuristic, presented above.

From Fig. 2a, it can be found that the median rapid response paths shaped by the lookahead search with the heuristic and the 1st generation *DNN*, respectively, are almost overlapping throughout the whole campaign, which proves that the trained *DNN* is able to interpret the nuanced variation of the system-level damage status of the *road network*, and mimic the decision-making of the *lookahead-based* strategy, resulting in a 10% increase on this strategy’s duration. Modest differences are also observed, in terms of *GWL*, which is the training target of the *DNN* (see “Methods” section), yielding the median values that are 12% higher than that associated with the *lookahead-based* strategy.

In view of the proven capability of the *DNN* with respect to the replication of the behaviour of the *span-based* heuristic, measured by both the duration and *GWL* associated with rapid responses, the effectiveness of the training paradigm proposed in this study has also been confirmed. Hence, compared to the pure *span-based* heuristic, the 1st generation *DNN* itself can now serve as a better experience generator, which is thereby employed to train a second generation of *DNN*. In other words, without domain (human) knowledge, the learning cycle itself is expected to push the performance boundary of the rapid response further, through granting the agent stronger planning capabilities.

Such an expectation is met, in light of the performance of the 2nd *generation DNN*, as shown in Fig. 2a and Table 1, which show a uniform 25% reduction (approximately) in the duration, compared to the baseline, across the median, 75%- and 95%-quantiles. In parallel, those three quantiles of *GWL* regarding such a *DNN* are found to yield a 11.5%, 18.5%, and 28.2% drop, respectively, with regard to those results of the 1st *generation* one. Therefore, these results have suggested that the performance of the rapid response associated with the *self-learnt*, 2nd generation *DNN* is able to coherently outperform its predecessor. More significantly, the more extreme the case is, the more pronounced the advantage obtained by the subsequent learning cycle could be.

Results in Figs. 2a and 3 show that the 3rd and 4th generation of the *DNN* do not improve further on the performance of the 2nd generation, indicating that the *DNN* generated through learnings has already “saturated” the performance of the corresponding strategy. Therefore, it further reveals that, with affordable computational costs, such a proposed strategy can autonomously scheme an optimal plan for a real-world, regional scale *RN* affected by disruptive events.

### Instantiation of the behaviour pattern of rapid responses guided by different strategies

**Figure 4:**
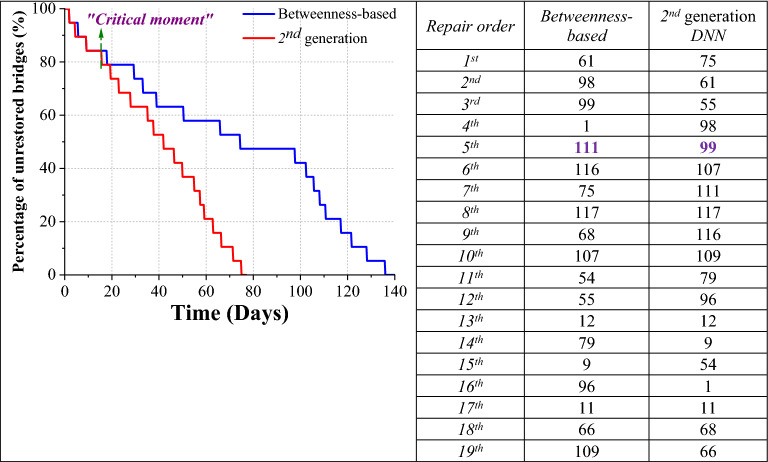
Post-shock rapid response shaped by the learning-based and the baseline strategies. The time-varying quantity of remaining severely-damaged bridges, which are under rapid responses following such two different strategies, are tracked. Especially, the “critical moment”, when the two trajectories bifurcated has also been highlighted.

To further discover how the *learning cycle* introduced above can enable the *rapid response agent* to outperform the previous strategies developed in this research, for the *learning-based strategy* (with the 2nd generation *DNN*) and the *betweenness-based* strategy (as the baseline), we examine their decisions throughout one particular realization of the simulation, where the initial damage status has already been presented in Fig. 1. Regarding the total of 19 severely-damaged bridges, the order in which they were sequenced by the two different strategies are traced and presented in Fig. 4, together with the resulting time-varying quantity thereof.

**Figure 5:**
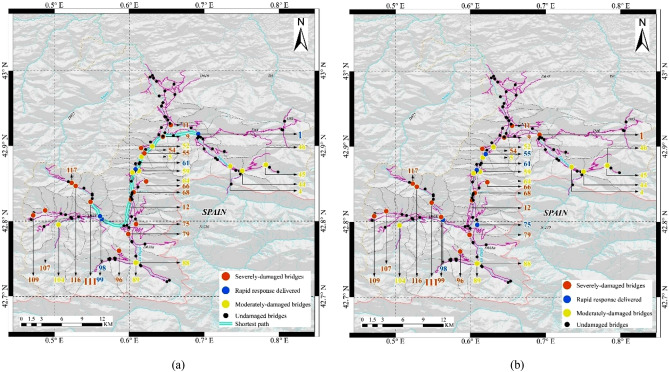
Global damage status of the *Road Network* at “critical” decision-moment. The plot is a “snapshot” of the damage status of the global *RN* at the 4th decision-moment of (**a**) the *betweenness-based strategy*, and (**b**) the *learning-based strategy*, respectively. The trajectory of the rapid response guided by those two different strategies significantly diverge thereafter, having a far-reaching impact on the whole campaign. The figures are plotted by ArcGIS.

Guided by those two different strategies, the decision-makings of the *agent* are found to be deviating from each other, from the very beginning of the rapid response campaign. Regarding the *betweenness-based* strategy, the *agent* will depart from the “City Centre” (see Fig. 1), which is also set to be the rapid response centre, to restore the *Bridge No*. 61, whose betweenness value is the highest among the 19 severely-damaged ones. By contrast, for the *learning-based strategy*, before the *Bridge No*. 61, the agent starts with the *Bridge No*. 75, which is very close to the *rapid response centre*, and therefore no other damaged bridge *en route*. It shall be highlighted that, until the completion of the rapid response of the 4th bridge (i.e. *No*. 98), such a strategy is indeed trailing behind the baseline, due to the different sequence of decision-makings*.* Nevertheless, as Fig. 4 reveals, since the “critical moment” that is the 16th day after the shock, the *learning-based strategy* not only starts to catch up with, but also outperform the *betweenness-based* one. Guided by the former, the *agent* will travel along the shortest path between *Bridges No.* 98 and *No.* 99, whose betweenness value is the 3rd highest. As shown in Fig. 5b, there are no unrepaired bridge along the path between those two bridges, indicating no delays in the delivery of the rapid response to that particular bridge. Similarly, following such a strategy, the *agent* will then continue to deliver rapid responses to the *Bridges No.* 107, *No.* 111, and *No.* 117, respectively, the connectivity among which is not disrupted by other still-damaged bridges either. Therefore, it takes only 19.25 days for the *agent* to complete the rapid response of all the four bridges listed above. As shown in Fig. 4, the betweenness value of the last three bridges are the 10th, 5th, and 8th highest, respectively, amidst the 19 ones. The prompt execution of their rapid responses is thereby crucial to the minimization of the *gross weighted losses*, that is the optimization objective of the strategy. Indeed, as highlighted in Figs. 4 and 5, the *agent* then continues by addressing *Bridges No.* 116, *No.* 109, and *No*. 79 (whose paths are not blocked, as well), re-establishing the connectivity of the whole southwest part of the *RN*, and thereby creating a clear shortest path between the valley and the epicentral area. The whole campaign clearing all the severely-damaged bridges takes a total of 75 days, with regard to such a strategy.

Conversely, informed by the *betweenness-based strategy*, the agent is directed to *Bridge No*. 111, after the completion of the rapid response of the Bridge *No*. 1. The shortest path between these two bridges is disrupted by a set of severely- and moderately-damaged bridges *en route* (see Fig. 5a), and the rapid response thus takes a substantially longer time. A similar situation is recurring during the whole sequence, with several long plateaux, due to the absence of adaptivity of this strategy. The overall duration of the rapid response is 136 days that is 1.8 times longer, compared to the *learning-based* one. Accordingly, the resulting *GWL* values are 1.9401e+03 and 1.1810e+03, for the two strategies, respectively.

## Discussion

We have proposed a “toolkit” consisting of *heuristic-*, *lookahead-*, and *learning-based* strategies of the post-hazard rapid response of large-scale critical infrastructure systems under disruptive events, as a pathway towards future hazard-resilient urban systems. The approach is illustrated by considering the post-earthquake recovery behaviour of road networks (*RNs*), where the partial repair on severely-damaged bridges under seismic hazards, is introduced as the rapid response. It has been highlighted that, following catastrophic earthquakes, it is extremely challenging to scheme an “optimal” rapid response plan of real-world *RNs*, when faced with widespread damage of various components, like bridges. Mathematically, the dimension of the search space grows exponentially with the amount of seismically-damaged bridges. Given the number of bridges subjected to the rapid response plan, denoted as *N*, there would then be a total of *N!* (approximately 2.43 × 10^18^, if *N* = 20) permutations of possible sequences, accordingly. Such a massive search space will render any brute-force paradigm computationally unaffordable and even unviable, especially, in the sense that rapid responses are inherently supposed to be planned and completed, in an expeditious and efficient pattern^6^.

As a simple alternative, we propose initially a *heuristic-based* strategy that is driven by a single and simple criterion, straightforward to implement and thereby feasible for stakeholders and decision-makers. The results show that the choice of the criterion does indeed play a decisive role with regard to the effectiveness of rapid responses. Specifically, when the bridge span that is essentially a measure on the ease of the repair, is considered to inform the strategy, our study shows that the delivery of rapid responses is not achievable in a reasonable timeframe with only one repair squad, as the path from one bridge to the next might be obstructed by bridges of larger span, or bridges needing minor repairs, especially, in the earlier phase of the campaign. This shows that a criterion based purely on component’s indicators is not sufficient to identify a valid strategy. By comparison, when the betweenness that is significantly more system-oriented, is employed as the criterion for the rapid response, the agent can complete the entire campaign, in nearly half the time. As the betweenness is indicative of the relevance of individual components, such a strategy can be assumed as the reference baseline for this class of problems.

However, the applicability of heuristic-based strategies to larger-scale infrastructure systems is limited by their lack of adaptability into the spatiotemporal evolution of the status of those systems throughout the post-hazard recovery phase. As an endeavour to remedy that deficiency, the lookahead-based method that allows to locally improve the strategy after each instantiation, is therefore proposed in this research. We have confirmed that, by introducing just one-step lookahead, which is often computationally tractable, the resulting rapid response, even based upon a component-oriented criterion, can still outcompete the baseline, both in terms of the *duration* and *gross weighted losses*. It thereby highlights the criticality of the review and reorientation (if necessary) of the sequential decision-making at each decision-moment throughout the rapid response campaign, when more agents are involved and their looped interaction is evolving as a result of the progress of their tasks, i.e. when the system or intertwined systems under recovery have a dynamic nature. The insight generated from the lookahead searches can be employed as an effective tool, to offset the potential losses of adaptivity, due to the limitations of the chosen heuristic criterion.

In view of the advantage associated with the lookahead searches, we managed to leverage the latest advances in *deep reinforcement learning*^14,23^ to enable the rapid response agent to keep pushing further the performance boundary autonomously, through an increasingly insightful understanding on the “value” of particular damage statuses of the global *CIS* of interest, with respect to the optimization objective. Hence, the third strategy in the *toolkit*, which marries *lookahead searches* with *deep reinforcement learning*, has been established and termed the *learning-based* strategy.

By training a total of four generation of *DNNs*, we have shown that, owing to the set of *hybrid features* delineating the damage status of the *RN* on both the *component-* and *system-*levels, the 1st generation *DNN*, that is trained based upon the *span-based* heuristic has enabled the replication of the behaviour of the *lookahead-based* strategy, where the same heuristic is incorporated. It is noteworthy that, similar to the case of *lookahead-based* strategy, we purposively choose to train the *DNN*, starting from such a naïve heuristic, to better fathom the self-learnt planning capability of the rapid response agent, without human knowledge. To this end, the following generations of *DNN* have all been trained from their own antecedent and are found to enable the rapid response performance surpassing that associated with the previous strategies in the *toolkit*, in a convincing way.

Furthermore, from one particular realization of the rapid response driven by the *learning-based strategy*, we further highlight that, owing to the planning capability granted by the *learning cycles*, the rapid response agent is able to balance the trade-off between the criticality and recoverability (which will further involve the connectivity pursuant to the real-time topology of the global network and the ease of the repair) of each single hazard-damaged component, in a sequential and adaptive way. Therefore, the agent could circumvent the encumbrances from the existence of the other damaged bridges throughout almost the entire campaign, by sometimes deliberately eschewing the harvest of the “low-hanging fruits”, which is the pattern of the corresponding *baseline* strategy. Hence, the *intelligence-demanding* objective of the *GWL* minimization has been fulfilled in such an approach, given the stark contrast between the performance of the rapid response driven by the two different strategies. Similar to the case of board games^24,25^, such a finding has also demonstrated that, through *learning*, the agent could autonomously explore the massive state space and complicated dynamics of real-world *CISs* throughout hazard events and outperform human experience-driven restorations. It can be thereby further concluded that, throughout the cycle where the better experience dataset will lead to better *DNNs*, which will, in turn, help to generate an even better experience dataset, the impact of the looped interdependence between the concurrent full-repair and the rapid response has also been better captured by the agent through the training.

To examine the consistency of the behavioural pattern of the proposed strategies that has been obtained regarding *Epicentre No.* 1 and discussed above, we apply them to scenarios generated on the basis of other epicentre locations (see *Epicentres No.* 2 and *No.* 3 in Fig. 1), which will therefore induce different spatial distributions of damage of the same *RN*. Seismic damage sustained by the *RN* tend to be less severe, as the epicentre location becomes farther from the city centre. Accordingly, the *GWL* associated with the corresponding rapid response campaign also decreases, in the case of the same strategy adopted. Nonetheless, the overall behaviour of the rapid response remains consistent with the one associated with *Epicentre No*. 1, as shown by the fitted probability density functions of *GWL*, in Supplementary Fig. 2. Besides, it can be noted that the performance of the *learning-based* strategy has been saturated after the 2nd generation *DNN*, as well, for both of those two epicentres. In particular, the reduction in the 95%-quantile of *GWL vis-à-vis* the *lookahead-* and the *learning-based* strategies compared to the baseline, are also close to the observed results regarding *Epicentre No.* 1. The fact that the performance of the *learning-based* strategies is independent of the epicentre location, indicates that the *pre-emptive* training of the *DNN* without necessitating sophisticated knowledge, is a viable option, which can override the inherent uncertainties of unpredictable hazard events^26^.

In light of the planning capabilities of the agent granted by the modelling framework established in this paper, the research is also relevant and crucial to the development of future, autonomous and *AI-capable* hazard recovery robots^27^, which can significantly reduce the risk exposure of human beings, across those inaccessible and inhabitable environments, in the wake of natural and/or man-made catastrophes^28^.

Meanwhile, it is also noteworthy that, the adaptivity of the rapid response is found to have a knock-on effect on the contemporaneous full repairs, which have been delayed paradoxically, as a consequence. Essentially, such an outcome suggests that the *rapid response agent*, whom we are training, has charted a pathway to fulfil the optimization goal, sometimes at the expense of its counterpart, who does not reorient its prioritization strategy in this study.

Finally, we would like to raise the question of the generality of the pattern associated with the findings of this research, in view of the possible discrepancy between the assumptions (e.g. the value of the behavioural attributes of the agents, as well as the physical model regarding the time needed by both the rapid response and full repair) made in this paper and the real-world cases. In parallel, it is also worthwhile to explore the scalability of the learning-based strategy developed in this study, when applied to significantly-larger *RNs* or some other *CISs*. It is certainly the case that the research is not able to give a definitive answer to those questions, nonetheless the consistency of the pattern of the results across different hazard scenarios, measured by the extremely high quantiles, does, we believe, lend some confidence to the applicability of the established modelling framework, when it comes to real-world *CISs*.

## Methods

### Agent-based model (*ABM*) on post-hazard rapid response of road networks

We examine the post-shock rapid response of *RNs*, through a multi agent-based model (*ABM*) established in this research, as a bottom-up and adaptive computational approach to the large-scale socio-economic systems^29,30^. In this study, only seismic damage of bridges in the *RN* will be considered, while the road segments have been assumed to be intact^31^. Under hazard events, the repair and/or reconstruction of severely-damaged bridges will often last several months^32–34^, and the resultingly longstanding connectivity losses of the *RN* will thus pose a grave challenge to the emergency transfer and the restoration of many other critical infrastructure systems, and ultimately, the resilience of the whole urban community, where such a *RN* is embedded^17^. Therefore, in this study, based on the damage they have sustained, those earthquake-damaged bridges will be divided into two groups, namely, the severely- and moderately-damaged ones, respectively. In particular, the partial repair, whose goal is to restore the functionality of those severely-damaged bridges to an incomplete, yet minimal acceptable level, has been characterized as the rapid response, in this research^6^. To account for and delineate the looped interdependence between the contemporaneous rapid responses and full repairs^6^, two agents, who are referred to as *Agent A* and *Agent B*, and set to be delivering the rapid response to those severely-damaged bridges, and the full repair to moderately-damaged ones, respectively, both in order, have been incorporated into the *ABM* established. It shall be highlighted that, the three different strategies proposed in this paper, namely, the *heuristic—*(see Note 1 in the Supplementary Information), the *lookahead-*, and the *learning-based* strategies, respectively, will all be employed to guide *Agent A*. Meanwhile, the strategy of *Agent B* will always be *betweenness-based*, regardless of the one adopted by its counterpart.

In this *ABM*, the rapid response will be modelled by two behavioural attributes of *Agent A*, namely, *E*_*s*_ and *V*_*s*_, which stand for its benchmark restoration efficiency, and the travel velocity, respectively. Similarly, the behaviour of *Agent B* will be tracked in the same way (but with an inherently lower recovery rate) as *Agent A* (except the reduction of its repair efficiency attribute, which is not modelled for such an agent), with two attributes, namely, *E*_*m*_ and *V*_*m*_, which refer to the efficiency and the travel velocity with regard to full repairs, respectively. In each of the realization of the simulation, the behaviour attributes of both the two agents will be sampled from uniform distributions, whose lower and upper bounds are presented in Table 2. Accordingly, to account for the collective impact of the uncertainty of the earthquake-initiated damage and the behavioural attributes of the agents on the system-level rapid responses, 1000 Monte Carlo simulations will be run for all the different strategies developed in this research.

**Table 2:** Behavioural attributes of the agents.

Agent	Attribute	Lower	Upper	Distribution	Average recovery time (days)
*A*	*V*_*s*_ (km/h)	15	20	Uniform	
	*E*_*s*_ (%)	50	100	Uniform	1.5
*B*	*V*_*m*_ (km/h)	5	10	Uniform	
	*E*_*m*_ (%)	5	10	Uniform	15

In this study, for both the *lookahead-* and *learning-based* strategies, the gross weighted loss (*GWL*) has been set as an inclusive optimization objective of *Agent A*. Regarding each of the realization of the rapid response campaign, *GWL* is computed following Eq. (1):

1 $$GWL = {\sum }_{t=0}^{{T}_{f}}\sum \limits_{k=1}^{{N}_{b}}{B}_{k}\times {RS}_{k,t}.$$

Here, *T*_*f*_ denotes the time point when the rapid response has been delivered to all the severely-damaged bridges. *B*_*k*_ and *RS*_*k,t*_ refer to the *betweenness* value of bridge *k* (*k* = 1, 2,…, *N*_*b*_, where *N*_*b*_ is the total number of bridges of the *road network* of interest), and the recovery state of such a bridge at the time point *t*, respectively. For each individual bridge, its recovery state can be 0, 1, 2 or 3, indicating totally recovered (or intact under the seismic hazard), minor damage but not yet recovered, severe damage and partially recovered, and severe damage but not yet recovered, respectively.

From the perspective of graph theory, the betweenness is one of the most important measures on the criticality of each single node, with regard to the connectivity of the corresponding network^35^. In this study, the betweenness centrality of bridge $k$, denoted as *B(k)*, will be computed following Eq. (2):

2 $$B\left(k\right)=\frac{\sum_{s\ne k\ne t}{\sigma }_{st}(k)}{{\sigma }_{tot}} .$$

Here ${\sigma }_{tot}$ refers to the total number of shortest paths associated with the whole *RN* of interest, while ${\sigma }_{st}(k)$ assume a value of 1, if the shortest path goes through *k*, and 0 otherwise.

If we assume a uniform distribution over all possible journeys within the network, *B*_*k*_ can be viewed as the probability of a random journey being affected by the damage to bridge *k*, while *RS*_*k,t*_ can be deemed as the measure on the level of the impact that recovery state has on that journey at time point *t*. Hence, *GWL* sums the expected impact of the state of the whole *RN* throughout the whole rapid response period, while taking account of the heterogeneity of the network. It can therefore be employed as an insightful measure on the total impact of the losses of the effective connectivity of the whole *RN*, and ultimately, the overall socio-economic losses incurred. Furthermore, given its proportional sensitivity to the bridge criticality, justifiably, a *betweenness-based* heuristic, which sequences all the severely-damaged bridges according to their betweenness value (in the descending order) can be spawned, which has been included in the *heuristic-based* strategies, as a greedy one and the baseline (for the other strategies in “toolkit” proposed in this research).

### Agent-based model on rapid responses guided by the lookahead-based strategy

The two *heuristic-based* strategies proposed in this study, i.e. the *span-* and *betweenness-based* ones (see Note 1 in the Supplementary Information), rely on an initial assessment and the rapid response sequence generated pursuant to a particular ranking criterion, without exploration on the time-varying state space of the *RN* due to both rapid responses and full repairs. In order to account for such evolutions, we have established a new strategy marrying the lookahead search with the heuristics mentioned above.

**Figure 6:**
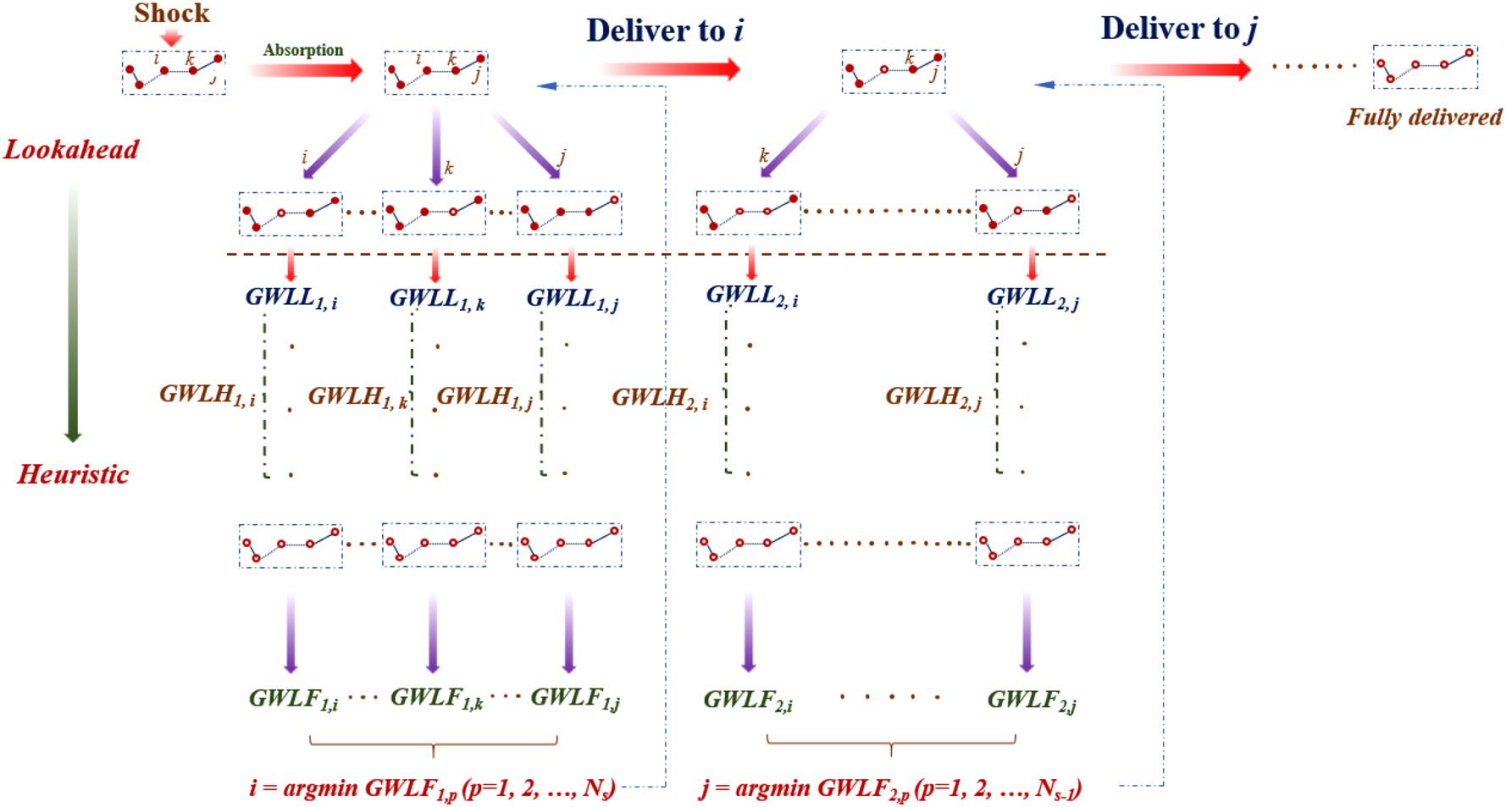
Lookahead-based strategy incorporating lookahead search and the heuristic. This figure illustrates how the lookahead searches can be run recursively, at each decision-moment, throughout the whole rapid response campaign. Essentially, driven by the combined outcome associated with the one-step lookahead, and the rollout of the succeeding heuristic, such a strategy is able to serve as the “move recommender” for the agent.

As shown in Fig. 6, supposedly, given *N*_*s*_ severely-damaged bridges immediately following one particular earthquake scenario, *Agent A* will first run a “virtual” one-step lookahead search, which can be described as follows.

For each bridge *p* $\in N$_*s*_, similar to Supplementary Eqs. (1)–(3), the corresponding time to complete its rapid response, denoted as *TL*_*1, p*_, can be obtained by Eq. (3):

3 $$ \begin{array}{*{20}l} {TL_{1,p} = T_{1,p} + \frac{{F_{r,p} }}{{E_{s} \times^{{n_{p} }} }},} \hfill & {{\text{if }}Span \left( p \right) \le { }50{\text{ m or}},} \hfill \\ {TL_{1,p} = T_{1,p} + \frac{{F_{r,p} }}{{E_{s} \times^{{n_{p} }} }} \times \left( {\frac{{{\text{Span}}\left( {\text{p}} \right)}}{50}} \right)^{2} ,} \hfill & {{\text{if }}Span\left( p \right) > { }50{\text{ m}}.} \hfill \\ \end{array} $$

Here, ${T}_{1,p}$ denotes the time needed for the rapid response crew to reach bridge *p*, while *F*_*r,p*_ stands for the functionality to be restored with regard to such a bridge. As explained in Note 1 in the Supplementary Information, *ω* (ω < 1) is a pre-defined reduction coefficient, while *n*_*p*_ stands for the number of unrepaired bridges standing on the shortest path, along which *Agent A* is travelling to tackle the next targeted bridge.

By replacing *T*_*f*_ with *TL*_*1, p*_, the resulting *GWL* associated with this one-step run, referred to as *GWLL*_*1, p*_, can be then computed according to Eq. (1). For each bridge *p*, starting from the time point *TL*_*1, p*_, as well as the corresponding *GWLL*_*1, p*_, *Agent A* will continue to deliver the rapid response to the remaining (*N*_*s*_ − 1) bridges, following the *span-based* heuristic. To that end, a particular rapid response sequence will be formulated, by ranking those (*N*_*s*_* − *1) bridges based on their spans, in the ascending order. For each individual bridge *q* in such a sequence, the resulting *GWL* throughout the loop of its rapid response, denoted as *GWL*_*1, p*_*(q)*, can be obtained following the Eq. (4):

4 $${GWL}_{1,p}\left(q\right)= \sum \limits_{t={TH}_{1,p}\left(q-1\right)}^{{TH}_{1,p}\left(q\right)}\sum \limits_{k=1}^{{N}_{b}}{B}_{k}\times {RS}_{k,t},$$

where *TH*_*1,p*_(*q*) and *TH*_*1,p*_(*q* − 1) stand for the time point when the rapid response has been delivered to the bridge *q* and the one before it, in that heuristic-generated sequence, respectively. In case of *q* = 1 (among those *Ns* − 1 ones), *TH*_*1,p*_(*q* − 1) = *TL*_*1, p*_. Until the rapid response has been delivered to all the (*N*_*s*_ − 1) bridges, the *GWL* associated with the heuristic rollout, referred to as *GWLH*_*1,p*_, will be computed following the Eq. (5):

5 $${GWLH}_{1,p}=\sum \limits_{\text{q}=1}^{{N}_{s}-1}{GWL}_{1,p}\left(q\right).$$

Accordingly, the *GWL vis-à-vis* the full rapid response sequence starting with the bridge *p*, denoted as *GWLF*_*1,p*_, can be obtained by aggerating the *GWLL*_*1, p*_ and *GWLH*_*1, p*_.

As revealed in Eq. (6), pursuant to the optimization objective, from the current state onward, the bridge *i*, associated with the particular trajectory among a total of *N*_*s*_ ones, which yields the minimum value of *GWL*, will be thereby picked by *Agent A*, as the targeted one.

6 $$i=\underset{p}\,{\text{arg min }}{GWLF}_{1,p}\left(p=1, 2, ..., {N}_{s}\right).$$

As illustrated in Fig. 6, at the next decision-moment, i.e. the time point when the rapid response of bridge *i* has been wrapped up, *Agent A* will pick the next targeted bridge, among the remaining ones, following the same decision-making loop that has been elaborated above. Therefore, the whole rapid response campaign will be shaped in such a recursive way, until its finalization with respect to all the *N*_*s*_ bridges.

### Agent-based model on rapid responses guided by the learning-based strategy

As explained above, the search following Eq. (6) will be driven by the outcome associated with the combined one-step lookahead and the following heuristic. However, given the inherently ad hoc nature of the damage status of the global infrastructure system under hazard events, the rollout of one particular, heuristic-generated rapid response sequence can very likely misinterpret the looped dynamics between the concurrent rapid response and the full repair.

Inspired by the latest breakthrough in the deep learning technique that is applicable to large-scale, complex networks^36–38^, we have trained a deep neural network (*DNN*), which generalizes the “past” experiences generated by massive amount of the hypothetical hazard scenarios, and can thereby “foresee” the expected losses onwards of the global infrastructure system, given a particular damage status thereof. Predicated upon its discernment, such a *DNN* can be employed to replace the rollout of the heuristic, and guide the lookahead searches.

To that end, without targeting any particular earthquake scenario, we will train the first generation of such a feedforward neural network, based upon the experiences generated from 8000 hypothetical earthquake scenarios with the epicentres uniformly located in the area with *latitude* ranging from 42.7° to 43° N, and *longitude* from 0.5° to 0.8° E (see Fig. 1). The magnitude of all of those scenarios have been set to be 7, to avoid trivial simulation outcomes.

As an attempt to better appraise the generalization potentiality of such a *learning-based* strategy, the initial *DNN* will be trained from the experiences generated following the *span-based* strategy, which has been proven to be significantly less effective than the betweenness-based one (i.e. the betweenness-based), with regard to all those 8000 earthquake scenarios.

**Figure 7:**
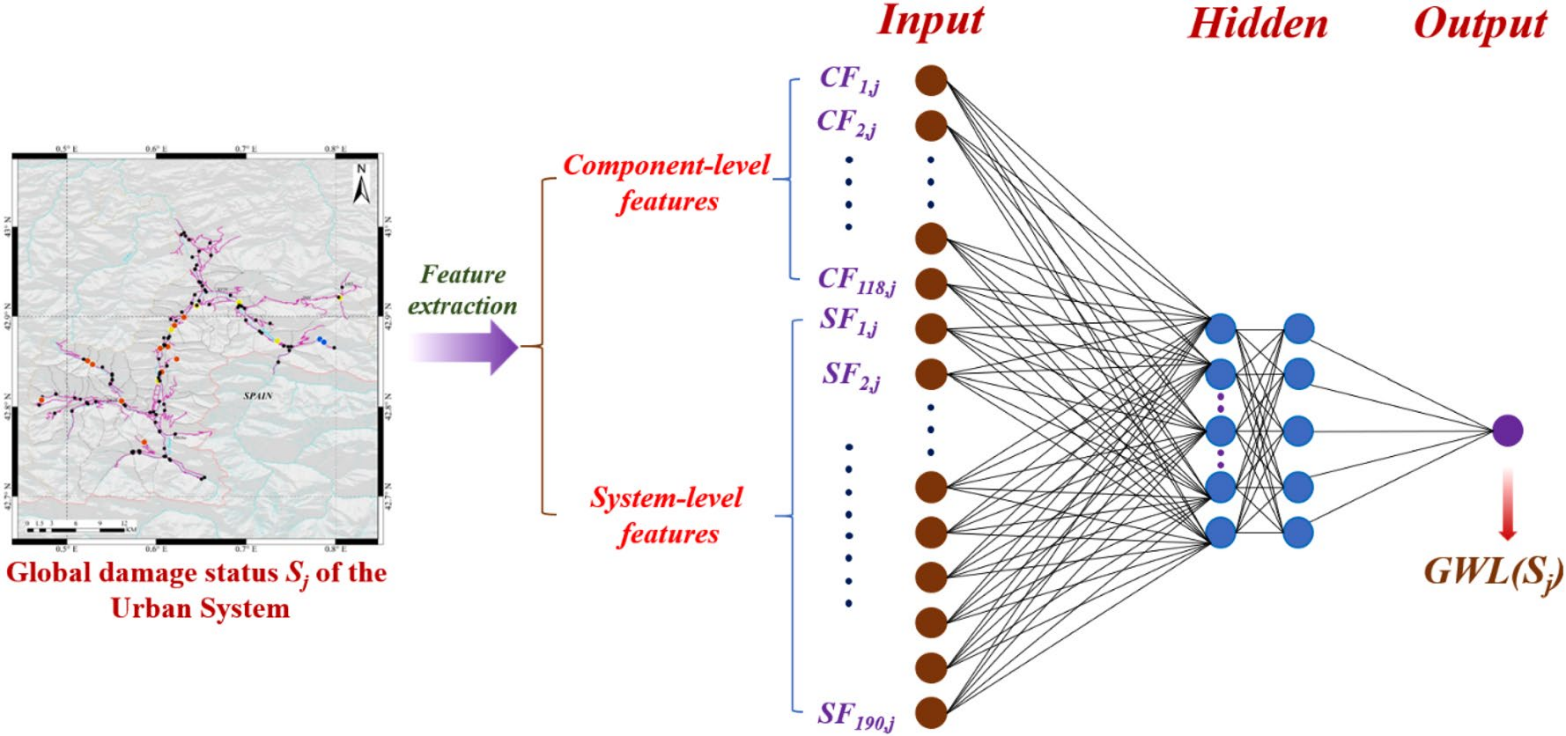
The architecture of the deep neural network. This plot illustrates how a “snapshot” of the damage status of the global *RN* can be translated into the set of “hybrid” features of the *DNN*, which can thereby foresee the corresponding *GWL*, from that status onwards. Besides, the *learning-based* strategy with the trained *DNN* will be employed to engender the experience dataset of better *DNNs* throughout training cycles, as well as the formation of rapid response strategies, when applied in the aftermath of real-world hazards. The map in the figure is plotted by ArcGIS.

For any single scenario leading to *N*_*s*_ ones of severely-damaged bridges, given the damage status of the global *RN* at the *j*th (*j* = 1, 2,…, *N*_*s*_) decision-moment, denoted as *S*_*j*_, the corresponding *GWL(S*_*j*_*)* will be obtained by aggregating all resulting *GWLs* onwards, following Eq. (1), until the end of the rapid response campaign. Particularly, to enable the *DNN* to better discover the mathematical link between each pair of the *S*_*j*_ and the corresponding *GWL(S*_*j*_*)* through the training, an array of the component- and system-level features will be generated, to translate *S*_*j*_ into the input of the *DNN*, as illustrated in Fig. 7.

Specifically, given the amount of bridges of the *RN* in *Luchon*, a total of 118 component-level features on the *j*th decision-moment, denoted as *CF*_*j, k*_, will be incorporated and quantified at first, following Eq. (7):

7 $$ CF_{j, \, k} = \, B_{k} \times RSj,_{k} (j = 1,2, \ldots ,N_{s} ; \, k = 1,2, \ldots ,118). $$

In parallel, the system-level features in this neural network will be characterized as the connectivity among the (fixed set of the) 20 most seismically-fragile bridges (plotted in Supplementary Fig. 3), at such a moment. Therefore, the dimension of the array of the system-level features will be 190.

Mathematically, let *m* equal to (*g* − 1) × 20* − *0.5 × (*g* − 1) × *g* + *h*, where *g* (= 1, 2,…, 19) and *h* (= *g* + 1, *g* + 2,…, 20) stands for the *g*th and *h*th bridges (among those 20 bridges), as the origin and the destination bridge, respectively, the corresponding system-level feature, referred to as *SF*_*j, m*_, will be determined according to Eq. (8):

8 $${SF}_{j,m}=\prod \limits_{l=1}^{{B}_{as}}{c}_{j, l }.$$

Here, *B*_*as*_ refers to the number of bridges associated with the shortest path between the *g*th and *h*th bridge, while *c*_*j,l*_ is the coefficient indicating the impact on the connectivity, pursuant to the recovery state of each of those bridges, on the *j*th decision-moment. Specifically, *C*_*j,l*_ would equal to 0.5, when the corresponding bridge remains either moderately-, or severely-damaged. Meanwhile, *C*_*j,l*_ value will be 0.75, when it comes to a severely-damaged bridge, where the rapid response has been delivered already.

Given the input and training target that have been obtained at the *j*th decision-moment, a particular batch of the experience, denoted as *E*_*j*_ = [*CF*_*j, k*;_ *SF*_*j, m*_| *GWL*(*S*_*j*_)] (*k* = 1, 2,…,118;* m* = 1, 2,…,190), will be established. Accordingly, based upon the whole rapid response campaign associated with such a particular scenario, a total of *N*_*s*_ batches of the experiences, can be therefore generated.

By integrating experiences generated pursuant to all those different 8000 scenarios, the total amount of batches of the experiences included in the entire training dataset is approximately 112,000, given the average amount of severely-damaged bridges equal to 14. Correspondingly, the architecture of the whole *DNN* will thereby comprise an input layer (308 × 1), two hidden layers (the dimension of both of which is 36 × 1), and an output layer (1 × 1), respectively, as illustrated in Fig. 7.

In such a learning-based strategy, for the *K*th (*K* ≥ 2) generation of the *DNN* with the same architecture shown in Fig. 7, their training dataset will be engendered by the one-step lookahead search married with the (*K* − 1)th network, instead of the span-based strategy employed, when it comes to the 1st generation one. Mathematically, in the course of the dataset production for the *K*th generation *DNN*, given a damage status (denoted as *S*_*j*_) of the whole *RN*, at the *j*th decision-moment, under an earthquake scenario inducing *N*_*s*_ ones of severely-damaged bridges, the next bridge for the rapid response will be selected following Eq. (9):

9 $$i=\underset{p}{\text{arg min }}{(GWLL}_{j,p}{+GWLP}_{K-1}({VS}_{j+1}\left(p\right))) (p=1, 2, ..., {N}_{s}-j+1).$$

Here, *GWLL*_*j,p*_ refers to the *GWL* associated with the one-step lookahead conditioned on the choice of the *p*th bridge, among the remaining (*N*_*s*_ − *j* + 1) ones. Meanwhile, *GWLP*_*K*−1_(*VS*_*j*+1_(*p*)) denotes the *GWL* predicted by the (*K* − 1)th generation of the *DNN*, associated with the “virtual state” on the (*j* + 1)th decision-moment, resulting from the “completion” of the rapid response of the *p*th bridge. In practice, the traning of the newer generation *DNNs* can be terminated, when the performace of the *learning-based* strategy has been stabilized (i.e. saturated). Finally, when applied to the same earthqauke scenraio, the resuting rapid response behvaior of such a strategy with the 1st generation *DNN* will be similar to that associated with the *lookaheasd-based* strategy, where decision-makings will be driven by the *span-based* heuristic (in addition to the preceding one-step lookahead), whom such a *DNN* has been trained to mimic.

## Supplementary Information

Supplementary Information.

## Data Availability

All data and models necessary to interpret, replicate and build upon the methods or findings reported in the article are available by L.S. (li.sun@ucl.ac.uk), upon request.
