# Supplementary material for: Artificial intelligence-informed planning for the rapid response of hazard-impacted road networks
Source: Sci Rep. 2022 Sep 29;12:16286. doi: 10.1038/s41598-022-19637-z (PMC9523040; doi:10.1038/s41598-022-19637-z)
Supplement: Supplementary file 1 — Supplementary Information. [file 41598_2022_19637_MOESM1_ESM.docx]

**Supplementary Information**

***Artificial Intelligence-informed planning for the Rapid Response of Hazard-impacted Road Networks***

L. Sun, J. Shawe-Taylor and D. D’Ayala

We further elaborated on the basic principles of the proposed modelling framework, while presenting some additional simulation outcome that are relevant to the findings highlighted in the *Main Body* of the paper. Accordingly, such a document will be organized as follows. The agent-based model on the post-shock rapid response of hazard-damaged *Road Networks* (*RNs*), following *heuristic-based* strategies, will be described in ***Supplementary Note 1***. The comparison between the simulation outcome and the corresponding fittings, in terms of the post-shock rapid response of the *RN* under the earthquake scenario with *Epicentre No.1*, will be discussed in ***Supplementary Note 2****.* Furthermore, ***Supplementary Note 3*** will present the simulation outcome with regard to *Epicentre Nos. 2* and *3*, to investigate the consistency of the behavioural pattern we have observed regarding *Epicentre No. 1*. In ***Supplementary Note 4***, we illustrate the geographic location of those *20* most fragile bridges, whereby the system-level features of the *Deep Neural Network* have been generated pursuant to the connectivity among them. Finally, the data on the span, typology, location, and fragility models for bridges included in the case-study *Road Network* is presented in ***Supplementary Note 5***.

**Note 1: Heuristic-based strategies of post-shock rapid response of *Road Networks***

We aim at proposing and examining the impact of different *heuristic-based* strategies on the rapidity and effectiveness of the post-shock rapid response of *RNs* under damaging earthquakes.

In this paper, two different heuristics, which are *span-* and *betweenness-*based, respectively, have been developed. In principle, the *span-based* heuristic, which prioritizes those bridges with shorter spans (which is a straightforward indicator of the repairability of bridges), is attempting to maximize the amount of severely-damaged bridges, where the partial repair has been delivered, given a particular time frame after the shock. By comparison, the *betweenness-based* heuristic will sequence the same set of bridges, based on their betweenness value, in the descending order. By doing so, the functionality of those bridges, which are most critical to the global connectivity of *RNs*, can be (partially) restored, at first.

Following the *ABM* proposed in this research, as shown in *Supplementary Figure 1*, in each of the simulation realization of the recovery of the *RN* throughout the rapid response campaign, the damage state (*DS*) of all the bridges will be determined at first, through seismic fragility analysis^1,2^, in the course of *Absorption* stage following the particular earthquake scenario^3^. In this study, the *DS* of a bridge is set to be *4* and *2*, in case of severe and moderate damage, respectively. Additionally, for those bridges that are intact, the corresponding *DSs* will be *1*.

Pursuant to the heuristic (*i.e.* either *span-* or *betweenness-*based) adopted, a sequence of all the severely-damaged bridges, which are planned to be partially repaired in order by *Agent A*, will be generated upon the start of the campaign immediately following the absorption stage. For the rapid response of each individual bridge *i* (out of a total of *N_s_* ones), to account for the influence of the other damaged bridges, the corresponding start and completion time, denoted as *T_r,i_* and *R_r,i_*, respectively, will be obtained following *Supplementary Equations (1-3)*:

*R_r,i_ =*$T_{r,i} +\frac{F_{r,i}}{E_{s}*{}^{n_{i}}}$, if *Span (i)* ≤ 50m or

*R_r,i_ =*$T_{r,i} +\frac{F_{r,i}}{E_{s}*{}^{n_{i}}}*{(\frac{\mathrm{Span}\left( i \right)}{50})}^{2}$, if *Span(i)* > 50m, (1)

with *i = 1: N_s_* and

*T_r,1_ =* ${{SD}_{r,0}}/{V_{s}}$ (2)

*T_r,i_ =* ${{SD}_{r,i}}/{V_{s}}$, *for i = 2: N_s_* (3)

where *T_r,1_* is the time needed for *Agent A* to start the rapid response of the first bridge in the generated sequence, since the shock. Given the *RN* topology, the travelling time between the *rapid response* centre and the *first bridge* is computed by the length of the shortest path between the two locations (referred to as *SD_r,0_*), divided by *V_s_*. Accordingly, the time needed to complete the rapid response of such a bridge, referred to as *R_r,1_,* will be thereby obtained by *T_r,1_* plus the corresponding repair time, as shown in *Supplementary Equation (1)*. It is noteworthy that, in such an *Equation*, *F_r,i_* stands for the functionality of those severely-damaged bridges to be restored by the rapid response activity, which in this study is set to be the regaining of accessibility that has been totally lost due to the seismic damage^4^.


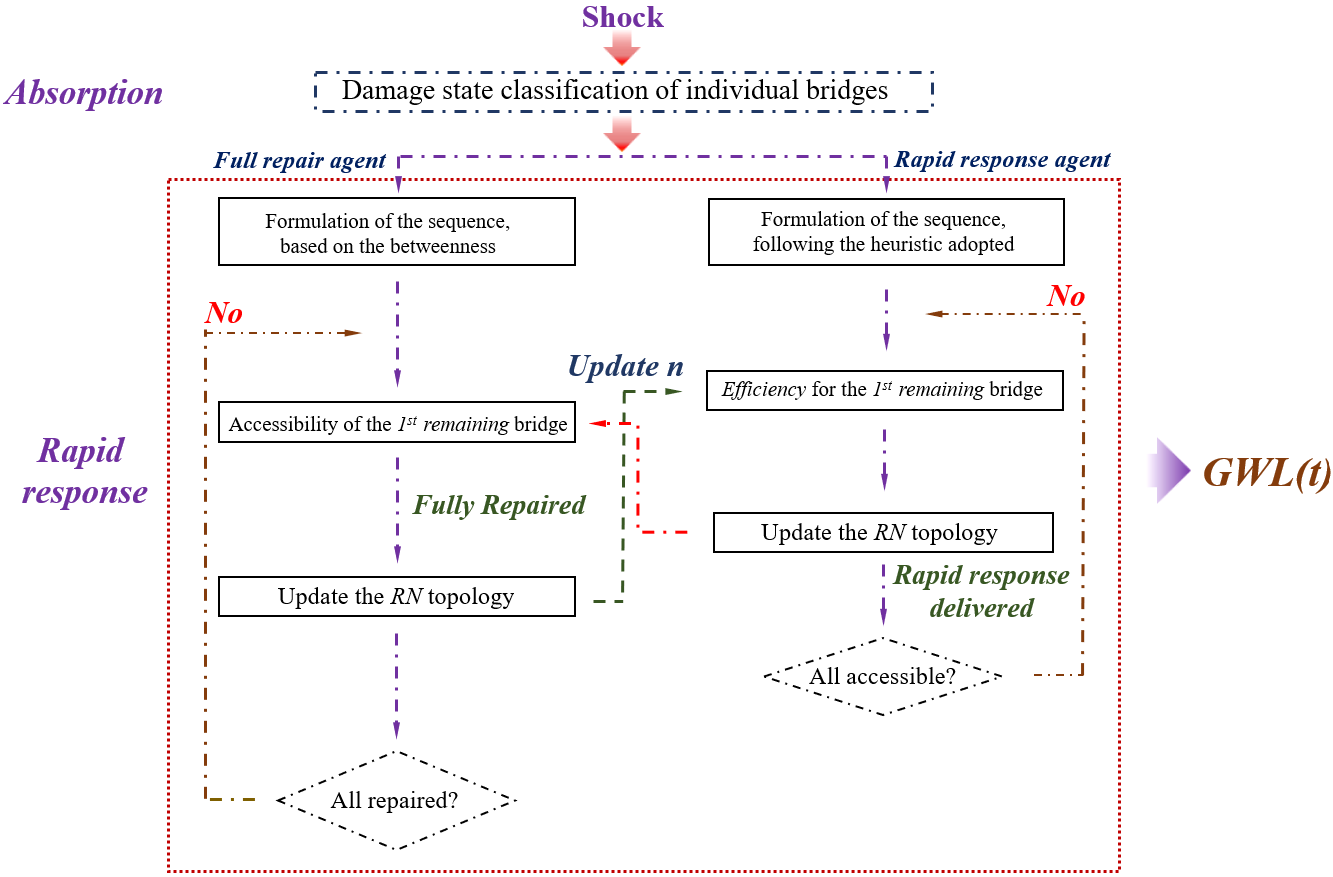


**Supplementary Figure 1. Modelling the recovery behaviour of *RNs*, shaped by the concurrent rapid response and full repair**. Two agents, who are in charge of the rapid response of severely-damaged bridges, and the concurrent full repair of moderately-damaged ones, respectively, have been incorporated.

In particular, as also shown in *Supplementary Equation (1),* the parameter of $E_{s}$ is set to be reduced by a factor $\omega^{n_{i}}$ (ω<1), where *ω* is a pre-defined reduction coefficient, while *n_i_* stands for the number of unrepaired bridges (include those severely-damaged, moderately-damaged, as well as partially repaired ones) associated with the shortest path between the last bridge that *Agent A* has delivered the rapid response, and the one it is heading to tackle. In this study, *ω* value is set to be 0.5, for those bridges with either severe, or moderate damage. However, for a severely-damaged bridge where the rapid response has been delivered already, the corresponding value *ω* will equal to 0.75. Therefore, predicted upon the amount and location of bridges that have been fully repaired by another agent or partially repaired by itself, the corresponding value of *n_i_* will be updated for the rapid response loop of bridge *i*, as shown in *Supplementary Figure 1*. Furthermore, for each individual bridge *i*, as revealed in *Supplementary Equation (1)*, its rapid response time will increase quadratically with *Span (i)*, if longer than 50m^5^.

In parallel, as also demonstrated in *Supplementary Figure 1*, the full repair of those moderately-damaged bridges, which will be sequenced based on their betweenness, is delivered by *Agent B* (with the two behavioural attributes, namely, *E_m_* and *V_m_*, respectively), in the course of the rapid response campaign. Nevertheless, it is noteworthy that, throughout its recovery endeavours, *Agent B* may encounter inaccessible paths, due to the presence of severely-damaged bridges. Regarding such cases, in this study, *Agent B* will wait, until the accessibility of those bridges occluding its available path forward has been restored, by its counterpart.

**Note 2: Additional simulation outcome regarding the earthquake scenario with *Epicentre No.1***

As mentioned in the *Main Body* of the paper, the probability density function following lognormal distributions have been generated to fit the *GWL* of the rapid response campaigns, associated with the simulation dataset, when different strategies proposed in this research have been adopted. To measure its accuracy, we calibrate the outcome of the fitting against the corresponding ones obtained from the simulation realizations. In particular, we are focusing upon the discrepancy between them, when it comes to the “extreme” cases. To that end, the *95%-*quantile of the *GWL* associated with the rapid response campaign under the earthquake scenario with *Epicentre No.1*, have been presented, in *Supplementary Table 1*. By comparison, it can be found that, the discrepancy rate of the fitted outcome has never exceeded *5.2%*, with regard to the corresponding simulations, suggesting that the generated probability density functions can match the simulation fairly well.

**Supplementary Table 1**. 95%-quantile of the *GWL* associated with the simulation dataset and the corresponding fittings (*10^3*)

|  | Baseline | Lookahead | *1^st^* generation | *2^nd^* generation | *3^rd^* generation | *4^th^* generation |
| --- | --- | --- | --- | --- | --- | --- |
| *Simulation* | 2.95 | 2.47 | 3.16 | 2.27 | 2.32 | 2.15 |
| *Fitting* | 2.89 | 2.39 | 3.00 | 2.25 | 2.20 | 2.17 |

**Note 3: Rapid response under the earthquake scenario with the other *Epicentres***

We further examined the consistency of the observed behavioural pattern of the post-shock rapid response under the earthquake scenario with *Epicentre No.1*, by applying the developed toolkit (of the whole host of rapid response strategies) to the *Luchon* *RN* under the earthquake scenarios with *Epicentres No. 2* and *3* (see *Figure 1*, in the *Main Body*), as well. Accordingly, the difference among the resulting rapid responses have been measured and presented in *Supplementary Figure 2*.

1. (b)

**Supplementary Figure 2**. ***GWL* of the post-shock rapid response under earthquake scenario with *Epicentre* *No.2* (*a*) and *Epicentre No.3* (*b*), and magnitude of *7.*** The probabilistic density functions have been fitted from the dataset of the resulting *GWL* of *1,000* realization of rapid response simulations, with all the strategies proposed.

Under both the *Epicentre Nos. 2* and *3*, it can be found that the overall behavioural pattern of the rapid response driven by different strategies have been rather consistent with those associated with *Epicentre No. 1*, as shown by the fitted probability density functions (all of which follow lognormal distribution). Hence, for the sake of brevity, we will be mainly focusing upon the distinction *vis-à-vis* the rapid responses following the *betweenness-*, the *lookahead-*, and the *learning*-based strategies (with the *2^nd^*, *3^rd^* and *4^th^* generation *DNN*), respectively, under both the two different *Epicentres*.

Quantitatively, regarding *GWL* under the scenario with *Epicentre No. 2*, the *95%-*quantile associated with the *lookahead-*, and the *learning-based* strategies (guided by those three *DNNs*) are *1.8682e+03*, *1.8648e+03*, *1.8758e+03* and *1.7633e+03*, leading to a reduction by *23.0%*, *23.1%*, *22.7%* and *27.3%*, respectively, compared to the baseline (*i.e.* *betweenness-based strategy*).

Similarly, when it comes to the *Epicentre No. 3* that is expected to induce the least damage, the corresponding value of the reduction associated with that array of strategies are found to reach *16.4%*, *15.3%*, *18.5%*, and *18.0%*, respectively.

**Note 4: The array of *20* bridges employed for the *system-level features* of the *DNN***


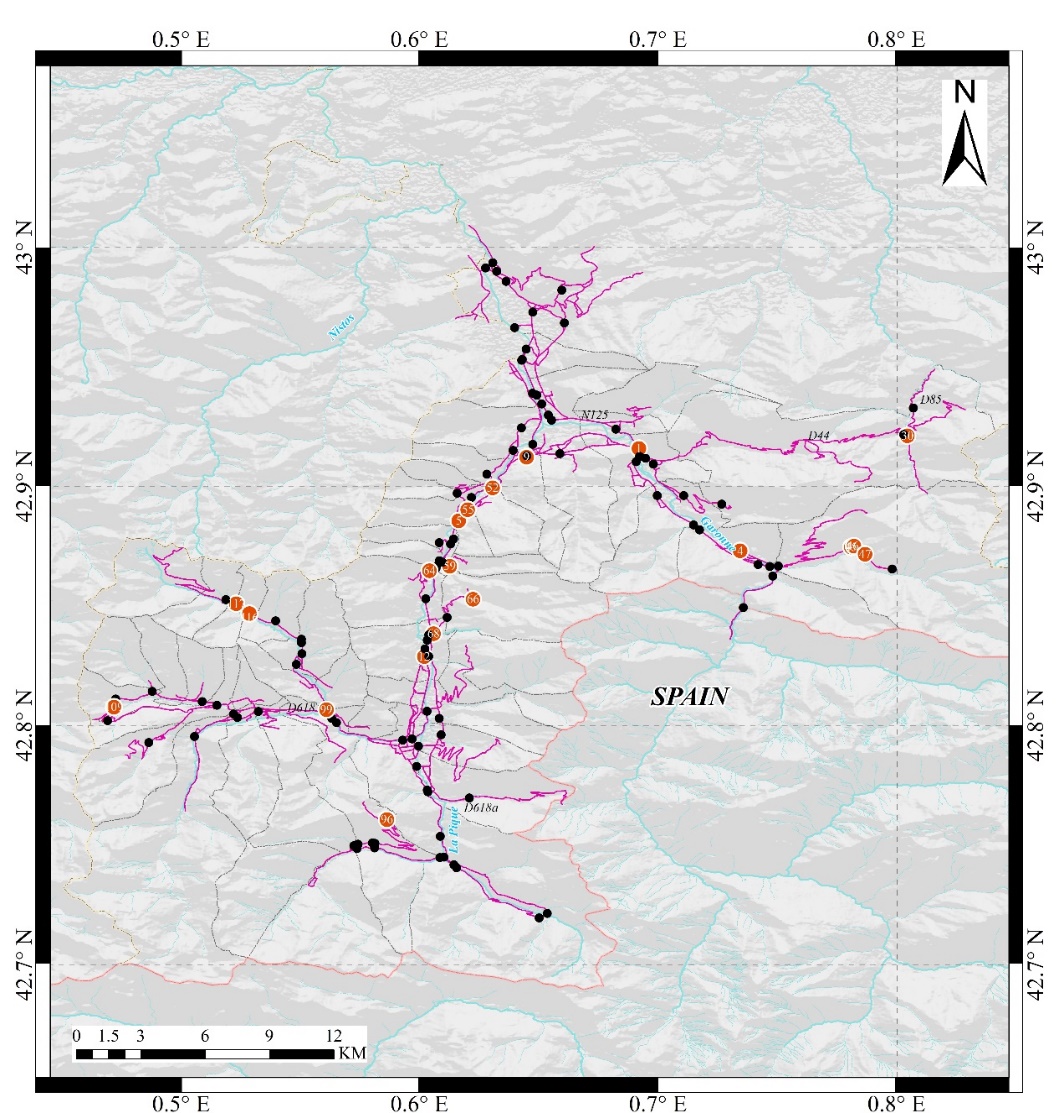


**Supplementary Figure 3**. **Geographic location of the *20* most seismically-fragile bridges of the *RN* in *Luchon***. The fragility behaviour of all the 118 bridges have been examined following the readily available fragility models^6,7^. The *20* most fragile bridges among them have been highlighted in orange colour. It can be found that, overall, they are “uniformly” distributed throughout the whole *RN*, which are therefore, sufficiently indicative of the real-time damage status thereof. The figure is plotted by ArcGIS.

**Note 5: Catalogue of bridges on the *Luchon* road network**

**Supplementary Table 2**. Data on the span, typology, location, and (lognormal) fragility models for bridges included in the *Luchon RN* (Source: *BRGM*, *France*)

| Bridge No. | Span (*m*) | Typology | Latitude (°) | Longitude (°) | Moderate damage | | Severe damage | |
| --- | --- | --- | --- | --- | --- | --- | --- | --- |
|  |  |  |  |  | *μ* | *σ* | *μ* | *σ* |
| 1 | 8.11 | Arch | 42.91565 | 0.691878 | 0.11 | 0.02 | 0.2 | 0.05 |
| 2 | 16.71 | 1-span beam | 42.82867 | 0.601152 | 1.447 | 0.897 | 1.753 | 1.086 |
| 3 | 111.84 | Truss | 42.96615 | 0.639766 | 0.575 | 0.328 | 0.656 | 0.374 |
| 4 | 33.53 | Arch | 42.8729 | 0.734417 | 0.09 | 0.02 | 0.2 | 0.05 |
| 5 | 11.47 | Arch | 42.88548 | 0.616425 | 0.09 | 0.02 | 0.2 | 0.05 |
| 6 | 2.82 | 1-span beam | 42.86725 | 0.741902 | 0.669 | 0.271 | 0.755 | 0.306 |
| 7 | 12.74 | 3-span beam | 42.95304 | 0.643001 | 1.166 | 1.123 | 1.61 | 1.551 |
| 8 | 12.8 | 3-span beam | 42.95259 | 0.642823 | 1.166 | 1.123 | 1.61 | 1.551 |
| 9 | 2.8 | Arch | 42.91214 | 0.644784 | 0.11 | 0.02 | 0.18 | 0.04 |
| 10 | 2.88 | 1-span beam | 42.91264 | 0.644899 | 1.447 | 0.897 | 1.753 | 1.086 |
| 11 | 12.88 | 3-span beam | 42.9276 | 0.655285 | 1.166 | 1.123 | 1.61 | 1.551 |
| 12 | 2.74 | Arch | 42.82869 | 0.601882 | 0.11 | 0.02 | 0.18 | 0.04 |
| 13 | 2.88 | 1-span beam | 42.86758 | 0.609411 | 1.447 | 0.897 | 1.753 | 1.086 |
| 14 | 7.79 | 1-span beam | 42.9934 | 0.630651 | 0.669 | 0.271 | 0.755 | 0.306 |
| 15 | 53.51 | 1-span beam | 42.99116 | 0.627592 | 0.669 | 0.271 | 0.755 | 0.306 |
| 16 | 12.09 | 1-span beam | 42.98979 | 0.63235 | 0.669 | 0.271 | 0.755 | 0.306 |
| 17 | 15.12 | 1-span beam | 42.98561 | 0.636202 | 0.669 | 0.271 | 0.755 | 0.306 |
| 18 | 10.62 | 1-span beam | 42.98191 | 0.659641 | 0.669 | 0.271 | 0.755 | 0.306 |
| 19 | 20.98 | 1-span beam | 42.97282 | 0.64737 | 0.669 | 0.271 | 0.755 | 0.306 |
| 20 | 5.84 | 1-span beam | 42.95727 | 0.644671 | 1.447 | 0.897 | 1.753 | 1.086 |
| 21 | 67.74 | 1-span beam | 42.93878 | 0.647311 | 0.79 | 0.787 | 1.524 | 1.518 |
| 22 | 22.48 | 1-span beam | 42.93798 | 0.649128 | 0.669 | 0.271 | 0.755 | 0.306 |
| 23 | 57.39 | 1-span beam | 42.93443 | 0.651228 | 0.669 | 0.271 | 0.755 | 0.306 |
| 24 | 25.11 | 1-span beam | 42.92971 | 0.654023 | 0.669 | 0.271 | 0.755 | 0.306 |
| 25 | 27.1 | 1-span beam | 42.92385 | 0.68226 | 0.669 | 0.271 | 0.755 | 0.306 |
| 26 | 41.03 | 2-span beam | 42.91236 | 0.692208 | 0.99 | 1.019 | 1.263 | 1.3 |
| 27 | 10.22 | 1-span beam | 42.91158 | 0.694663 | 0.669 | 0.271 | 0.755 | 0.306 |
| 28 | 8.51 | 1-span beam | 42.91023 | 0.69078 | 1.447 | 0.897 | 1.753 | 1.086 |
| 29 | 8.84 | 1-span beam | 42.90918 | 0.697997 | 0.669 | 0.271 | 0.755 | 0.306 |
| 30 | 40.19 | Arch | 42.92101 | 0.804543 | 0.15 | 0.03 | 0.32 | 0.08 |
| 31 | 17.95 | 1-span beam | 42.92164 | 0.803035 | 0.669 | 0.271 | 0.755 | 0.306 |
| 32 | 13.49 | 1-span beam | 42.93266 | 0.807032 | 1.447 | 0.897 | 1.753 | 1.086 |
| 33 | 8.88 | 1-span beam | 42.92435 | 0.642655 | 0.669 | 0.271 | 0.755 | 0.306 |
| 34 | 49.86 | 1-span beam | 42.91737 | 0.647403 | 0.669 | 0.271 | 0.755 | 0.306 |
| 35 | 23.85 | 1-span beam | 42.91495 | 0.63919 | 0.669 | 0.271 | 0.755 | 0.306 |
| 36 | 12.06 | 1-span beam | 42.91362 | 0.658803 | 1.447 | 0.897 | 1.753 | 1.086 |
| 37 | 13.52 | 1-span beam | 42.89597 | 0.710659 | 0.669 | 0.271 | 0.755 | 0.306 |
| 38 | 4.54 | 1-span beam | 42.89247 | 0.726729 | 0.669 | 0.271 | 0.755 | 0.306 |
| 39 | 13.81 | 1-span beam | 42.896 | 0.699634 | 0.669 | 0.271 | 0.755 | 0.306 |
| 40 | 32.77 | 1-span beam | 42.88381 | 0.714911 | 0.669 | 0.271 | 0.755 | 0.306 |
| 41 | 9.46 | 1-span beam | 42.88171 | 0.71742 | 0.669 | 0.271 | 0.755 | 0.306 |
| 42 | 11.33 | 1-span beam | 42.9682 | 0.660713 | 1.447 | 0.897 | 1.753 | 1.086 |
| 43 | 127.6 | 1-span beam | 42.86635 | 0.746817 | 0.79 | 0.787 | 1.524 | 1.518 |
| 44 | 17.19 | Arch | 42.86655 | 0.750375 | 0.28 | 0.13 | 0.45 | 0.23 |
| 45 | 12.11 | Arch | 42.87452 | 0.780903 | 0.09 | 0.02 | 0.2 | 0.05 |
| 46 | 16.74 | Arch | 42.87493 | 0.78207 | 0.09 | 0.02 | 0.2 | 0.05 |
| 47 | 17.23 | Arch | 42.87148 | 0.786771 | 0.09 | 0.02 | 0.2 | 0.05 |
| 48 | 3.37 | 1-span beam | 42.86533 | 0.798089 | 1.447 | 0.897 | 1.753 | 1.086 |
| 49 | 12.88 | 1-span beam | 42.86237 | 0.748101 | 0.669 | 0.271 | 0.755 | 0.306 |
| 50 | 83.02 | 1-span beam | 42.84921 | 0.735701 | 0.79 | 0.787 | 1.524 | 1.518 |
| 51 | 20.6 | 1-span beam | 42.905 | 0.628191 | 0.669 | 0.271 | 0.755 | 0.306 |
| 52 | 15.77 | Arch | 42.89929 | 0.630572 | 0.09 | 0.02 | 0.2 | 0.05 |
| 53 | 18.91 | 1-span beam | 42.8952 | 0.621745 | 0.669 | 0.271 | 0.755 | 0.306 |
| 54 | 15.02 | 1-span beam | 42.89696 | 0.615715 | 0.669 | 0.271 | 0.755 | 0.306 |
| 55 | 10.47 | Arch | 42.89007 | 0.620103 | 0.09 | 0.02 | 0.2 | 0.05 |
| 56 | 103.45 | 3-span beam | 42.87791 | 0.614184 | 0.575 | 0.328 | 0.656 | 0.374 |
| 57 | 29.23 | 2-span beam | 42.87577 | 0.612961 | 0.99 | 1.019 | 1.263 | 1.3 |
| 58 | 9.14 | 1-span beam | 42.87629 | 0.608223 | 0.669 | 0.271 | 0.755 | 0.306 |
| 59 | 29.04 | Arch | 42.86651 | 0.612568 | 0.15 | 0.03 | 0.32 | 0.08 |
| 60 | 28.73 | 1-span beam | 42.86817 | 0.609637 | 0.669 | 0.271 | 0.755 | 0.306 |
| 61 | 9.98 | 1-span beam | 42.86876 | 0.608293 | 1.447 | 0.897 | 1.753 | 1.086 |
| 62 | 23.78 | 1-span beam | 42.86612 | 0.606288 | 0.669 | 0.271 | 0.755 | 0.306 |
| 63 | 25.13 | 1-span beam | 42.86559 | 0.606916 | 0.669 | 0.271 | 0.755 | 0.306 |
| 64 | 40.17 | Arch | 42.8646 | 0.60411 | 0.15 | 0.03 | 0.32 | 0.08 |
| 65 | 31.49 | 1-span beam | 42.85285 | 0.602605 | 0.669 | 0.271 | 0.755 | 0.306 |
| 66 | 17.93 | Arch | 42.85267 | 0.622411 | 0.16 | 0.03 | 0.27 | 0.07 |
| 67 | 13.23 | 1-span beam | 42.84509 | 0.611486 | 1.447 | 0.897 | 1.753 | 1.086 |
| 68 | 21.25 | Arch | 42.83826 | 0.605814 | 0.15 | 0.03 | 0.32 | 0.08 |
| 69 | 10.65 | 1-span beam | 42.83778 | 0.603807 | 0.669 | 0.271 | 0.755 | 0.306 |
| 70 | 11.79 | 1-span beam | 42.83559 | 0.603212 | 0.669 | 0.271 | 0.755 | 0.306 |
| 71 | 34.57 | 1-span beam | 42.83189 | 0.602151 | 0.669 | 0.271 | 0.755 | 0.306 |
| 72 | 22.05 | 1-span beam | 42.82876 | 0.603765 | 0.669 | 0.271 | 0.755 | 0.306 |
| 73 | 97.78 | 1-span beam | 42.8059 | 0.60315 | 0.79 | 0.787 | 1.524 | 1.518 |
| 74 | 16.35 | 1-span beam | 42.80295 | 0.608234 | 1.447 | 0.897 | 1.753 | 1.086 |
| 75 | 30.28 | 1-span beam | 42.79596 | 0.608965 | 0.669 | 0.271 | 0.755 | 0.306 |
| 76 | 17.62 | 1-span beam | 42.79434 | 0.596788 | 1.447 | 0.897 | 1.753 | 1.086 |
| 77 | 27.16 | 1-span beam | 42.79383 | 0.592899 | 0.669 | 0.271 | 0.755 | 0.306 |
| 78 | 29.7 | 1-span beam | 42.79135 | 0.599499 | 0.669 | 0.271 | 0.755 | 0.306 |
| 79 | 24.01 | 1-span beam | 42.78277 | 0.598704 | 0.669 | 0.271 | 0.755 | 0.306 |
| 80 | 10.94 | 1-span beam | 42.77285 | 0.603205 | 0.669 | 0.271 | 0.755 | 0.306 |
| 81 | 6.12 | 1-span beam | 42.77216 | 0.603537 | 0.669 | 0.271 | 0.755 | 0.306 |
| 82 | 18.02 | 1-span beam | 42.76964 | 0.620848 | 1.447 | 0.897 | 1.753 | 1.086 |
| 83 | 85.24 | 1-span beam | 42.75361 | 0.608748 | 0.79 | 0.787 | 1.524 | 1.518 |
| 84 | 57.25 | 1-span beam | 42.74168 | 0.614449 | 1.447 | 0.897 | 1.753 | 1.086 |
| 85 | 15.06 | 1-span beam | 42.74037 | 0.615521 | 1.447 | 0.897 | 1.753 | 1.086 |
| 86 | 23.77 | 1-span beam | 42.71952 | 0.650138 | 1.447 | 0.897 | 1.753 | 1.086 |
| 87 | 49.81 | 1-span beam | 42.72132 | 0.653524 | 1.447 | 0.897 | 1.753 | 1.086 |
| 88 | 39.05 | Arch | 42.74492 | 0.610161 | 0.15 | 0.03 | 0.32 | 0.08 |
| 89 | 56.26 | Arch | 42.74474 | 0.608483 | 0.15 | 0.03 | 0.32 | 0.08 |
| 90 | 13.81 | 1-span beam | 42.74867 | 0.581132 | 0.669 | 0.271 | 0.755 | 0.306 |
| 91 | 17.19 | 1-span beam | 42.74854 | 0.573684 | 0.669 | 0.271 | 0.755 | 0.306 |
| 92 | 31.47 | 1-span beam | 42.74956 | 0.572367 | 0.669 | 0.271 | 0.755 | 0.306 |
| 93 | 34.81 | 1-span beam | 42.75034 | 0.574208 | 0.669 | 0.271 | 0.755 | 0.306 |
| 94 | 19.75 | 1-span beam | 42.75079 | 0.580247 | 1.447 | 0.897 | 1.753 | 1.086 |
| 95 | 13.96 | 1-span beam | 42.75056 | 0.581312 | 0.669 | 0.271 | 0.755 | 0.306 |
| 96 | 12.59 | Arch | 42.76063 | 0.586301 | 0.16 | 0.03 | 0.27 | 0.07 |
| 97 | 42.77 | 1-span beam | 42.80108 | 0.565141 | 0.669 | 0.271 | 0.755 | 0.306 |
| 98 | 38.09 | 1-span beam | 42.80281 | 0.563184 | 0.669 | 0.271 | 0.755 | 0.306 |
| 99 | 14.12 | Arch | 42.80649 | 0.560939 | 0.16 | 0.03 | 0.27 | 0.07 |
| 100 | 10.64 | 1-span beam | 42.80584 | 0.532401 | 0.669 | 0.271 | 0.755 | 0.306 |
| 101 | 21.21 | 1-span beam | 42.80326 | 0.523586 | 0.669 | 0.271 | 0.755 | 0.306 |
| 102 | 42.91 | 1-span beam | 42.80469 | 0.521998 | 0.669 | 0.271 | 0.755 | 0.306 |
| 103 | 22.99 | 1-span beam | 42.80843 | 0.51496 | 0.669 | 0.271 | 0.755 | 0.306 |
| 104 | 28.49 | Arch | 42.79531 | 0.505596 | 0.15 | 0.03 | 0.32 | 0.08 |
| 105 | 10 | 1-span beam | 42.79282 | 0.486378 | 0.669 | 0.271 | 0.755 | 0.306 |
| 106 | 25.84 | 1-span beam | 42.80991 | 0.508834 | 0.669 | 0.271 | 0.755 | 0.306 |
| 107 | 31.66 | Arch | 42.8142 | 0.487941 | 0.15 | 0.03 | 0.32 | 0.08 |
| 108 | 25.65 | Arch | 42.81098 | 0.472603 | 0.15 | 0.03 | 0.32 | 0.08 |
| 109 | 8.99 | Arch | 42.80755 | 0.471846 | 0.19 | 0.05 | 0.3 | 0.07 |
| 110 | 12.27 | 1-span beam | 42.80193 | 0.469205 | 0.669 | 0.271 | 0.755 | 0.306 |
| 111 | 22.24 | Arch | 42.82547 | 0.548263 | 0.15 | 0.03 | 0.32 | 0.08 |
| 112 | 9.35 | 1-span beam | 42.83001 | 0.550554 | 0.669 | 0.271 | 0.755 | 0.306 |
| 113 | 8.12 | 1-span beam | 42.83454 | 0.550508 | 0.669 | 0.271 | 0.755 | 0.306 |
| 114 | 14.11 | 1-span beam | 42.836 | 0.550471 | 0.669 | 0.271 | 0.755 | 0.306 |
| 115 | 15.76 | 1-span beam | 42.84371 | 0.539565 | 0.669 | 0.271 | 0.755 | 0.306 |
| 116 | 16.03 | Arch | 42.84669 | 0.52864 | 0.09 | 0.02 | 0.2 | 0.05 |
| 117 | 16.07 | Arch | 42.85081 | 0.523081 | 0.09 | 0.02 | 0.2 | 0.05 |
| 118 | 21.13 | Arch | 42.85249 | 0.518821 | 0.15 | 0.03 | 0.32 | 0.08 |

**References**

## Mackie, K. R., and Stojadinovic, B. (2006). Post-earthquake functionality of highway overpass bridges. *Earthquake Engineering & Structural Dynamics* 35(1): 77-93.

## Gehl, P., and D'Ayala, D. (2018). System loss assessment of bridge networks accounting for multi-hazard interactions. *Structure and Infrastructure Engineering* 14(10): 1355-1371.

1. Ouyang, M., Dueñas-Osorio, L., and Min, X. (2012). A three-stage resilience analysis framework for urban infrastructure systems. *Structural Safety* 36-37: 23-31.
2. Sun, L., D’Ayala, D., Fayjaloun, R., and Gehl, P. (2021). Agent-based model on resilience-oriented rapid responses of road networks under seismic hazard. *Reliability Engineering and System Safety* 216: 108030.
3. Yeh, F. Y., Chang, K. C., Sung, Y. C., Hung, H. H., and Chou, C. C. (2015). A novel composite bridge for emergency disaster relief: Concept and verification. *Composite Structures* 127: 199-210.
4. Shinozuka, M., Feng, M. Q., Kim, H. K., Uzawa, T., and Ueda, T. (2003). *Statistical analysis of fragility curves*. Technical report MCEER-03-0002. [*http://shinozuka.eng.uci.edu/Pdf/RepFrag.pdf*](http://shinozuka.eng.uci.edu/Pdf/RepFrag.pdf)
5. Zampieri, P. (2014). *Simplified seismic vulnerability assessment of masonry arch bridges*. Ph.D. Thesis, University of Trento, Italy.
